# CT-based subregional and peritumoral radiomics for predicting pathological T stage of clear cell renal cell carcinoma: an exploratory study of biological mechanisms

**DOI:** 10.1186/s13244-026-02226-3

**Published:** 2026-02-16

**Authors:** Jun-Lin Huang, Qiao Liu, Cheng-Long Wang, Xuan Lang, Yu-Xi Zeng, Dai-Quan Zhou

**Affiliations:** 1 Department of Radiology, The Third Affiliated Hospital of Chongqing Medical University (Fangda Hospital), Chongqing, China; 2 Department of Radiology, West China School of Public Health and West China Fourth Hospital, Sichuan University, Chengdu, China

**Keywords:** Clear cell renal cell carcinoma, Pathological T stage, Radiomics, Machine learning, Biological mechanisms

## Abstract

**Objectives:**

To evaluate intratumoral subregional and peritumoral radiomics for predicting pathological T stage of clear cell renal cell carcinoma (ccRCC), and investigate the biological mechanisms of radiomics.

**Materials and methods:**

This retrospective study included 323 ccRCC patients from two centers, divided into training (*n* = 148), internal test (*n* = 38), and external validation (*n* = 137) sets. Patients were stratified into low (T1 and T2, *n* = 222) and high (T3 and T4, *n* = 101) T stage groups. The tumors were segmented into different intratumoral subregions via the Gaussian mixture model (GMM). Radiomic features (RFs) were extracted from the whole tumor region (VOI_whole), intratumoral subregions (VOI_subx), and the peritumoral region (VOI_peri). Several machine learning (ML) models and radiomic score (Radscore) were developed to predict pathological T stage and prognosis of ccRCC. Radiogenomics analysis was used to explore the relationship between radiomics and biologic pathways.

**Results:**

Two intratumoral subregions were segmented. The support vector machine (SVM)-based combined model, constructed using RFs from VOI_sub1 and VOI_peri, achieved the highest AUC values, of 0.82 (95% CI: 0.68–0.96) and 0.80 (95% CI: 0.71–0.88) in the internal test and external validation sets, respectively. A higher Radscore was correlated with poorer overall survival (OS) (*p* < 0.001). Radiogenomics analysis revealed that radiomics was associated with extracellular matrix remodeling, vesicle transport, protein processing in the endoplasmic reticulum, and the Hippo signaling pathway.

**Conclusions:**

An ML model combining intratumoral subregion and peritumoral RFs showed good performance in predicting the pathological T stage of ccRCC, and these RFs were associated with biological pathways underlying tumor invasion.

**Critical relevance statement:**

This study develops a validated CT-radiomics model (intratumoral subregions + peritumoral) predicting ccRCC T stage. The prognostic Radscore links to invasion biology (ECM remodeling, Hippo/ER dysregulation), enabling clinical translation.

**Key Points:**

- Subregional and peritumoral radiomics models accurately predicted ccRCC (clear cell renal cell carcinoma) histological T stage.
- Radiomics score identified that high-risk ccRCC patients had poorer overall survival.
- Predictive radiomic features (RFs) were associated with biological pathways underlying tumor invasion.

**Graphical Abstract:**

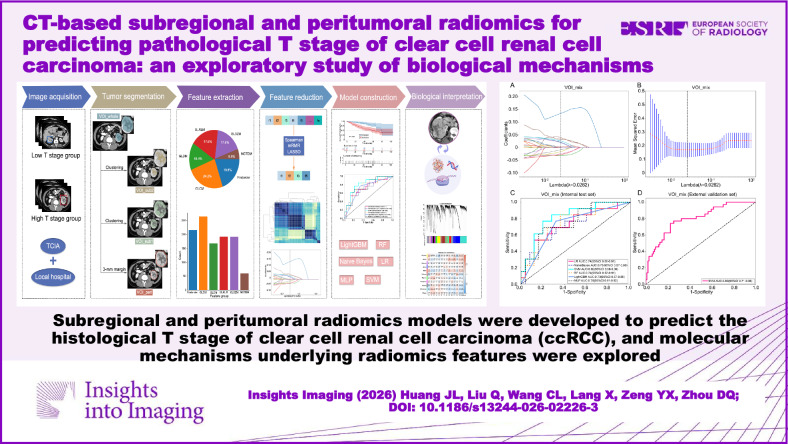

## Introduction

Renal cell carcinoma (RCC) ranks among the most common urological malignancies worldwide, accounting for 3–4% of all cancer diagnoses and contributing to approximately 3% of global cancer-related mortality [1, 2]. Clear cell renal cell carcinoma (ccRCC), representing 70–85% of RCC cases, exhibits aggressive biological behavior with a 5-year survival rate below 50% and substantially higher metastatic potential compared to other RCC subtypes [3, 4]. These statistics underscore the critical need for accurate prognostic stratification to guide clinical decision-making.

Current prognostic stratification in ccRCC relies principally on histological grading and the TNM staging system, maintained by the American Joint Committee on Cancer (AJCC) and the Union for International Cancer Control (UICC) [5]. While postoperative pathological staging based on surgical specimens provides robust prognostic information, preoperative staging remains challenging. Conventional approaches combine clinical imaging (e.g., CT) with invasive biopsies, which carry risks of hemorrhage, infection, and needle tract seeding, while suffering from sampling errors and inter-observer variability [6, 7]. Moreover, traditional CT imaging demonstrates limited accuracy in distinguishing organ-confined tumors (T1/T2) from those with renal vein invasion or perinephric fat involvement (T3a), due to mimics such as inflammatory changes or pseudocapsule formation [8, 9]. Even tumor size, a cornerstone of staging, becomes an unreliable predictor beyond T3 stages [10, 11]. Collectively, these limitations highlight an unmet need for non-invasive, biologically informed staging tools.

The emergence of radiomics has introduced a paradigm shift in oncological imaging by enabling high-throughput extraction of quantitative features from medical images to characterize tumor phenotypes and heterogeneity [11, 12]. Although radiomics shows promise in predicting tumor subtype, grade, TNM stage and prognosis [13, 14], existing studies predominantly rely on whole-tumor segmentation, which may obscure critical intratumoral spatial heterogeneity. Furthermore, the biological underpinnings of radiomic features (RFs) remain poorly understood, limiting their clinical interpretability.

To address these gaps, our study proposes two key innovations: first, the combination of intratumoral subregional radiomics and peritumoral radiomics to construct predictive models; and second, the integration of radiogenomics analysis through correlating RFs with transcriptomic data to elucidate the underlying biological mechanisms of radiomics. Through this approach, we aimed to develop a non-invasive predictive model for preoperative pathological T stage and uncover the biological significance of radiomics.

## Materials and methods

### Patients

**Fig. 1:**
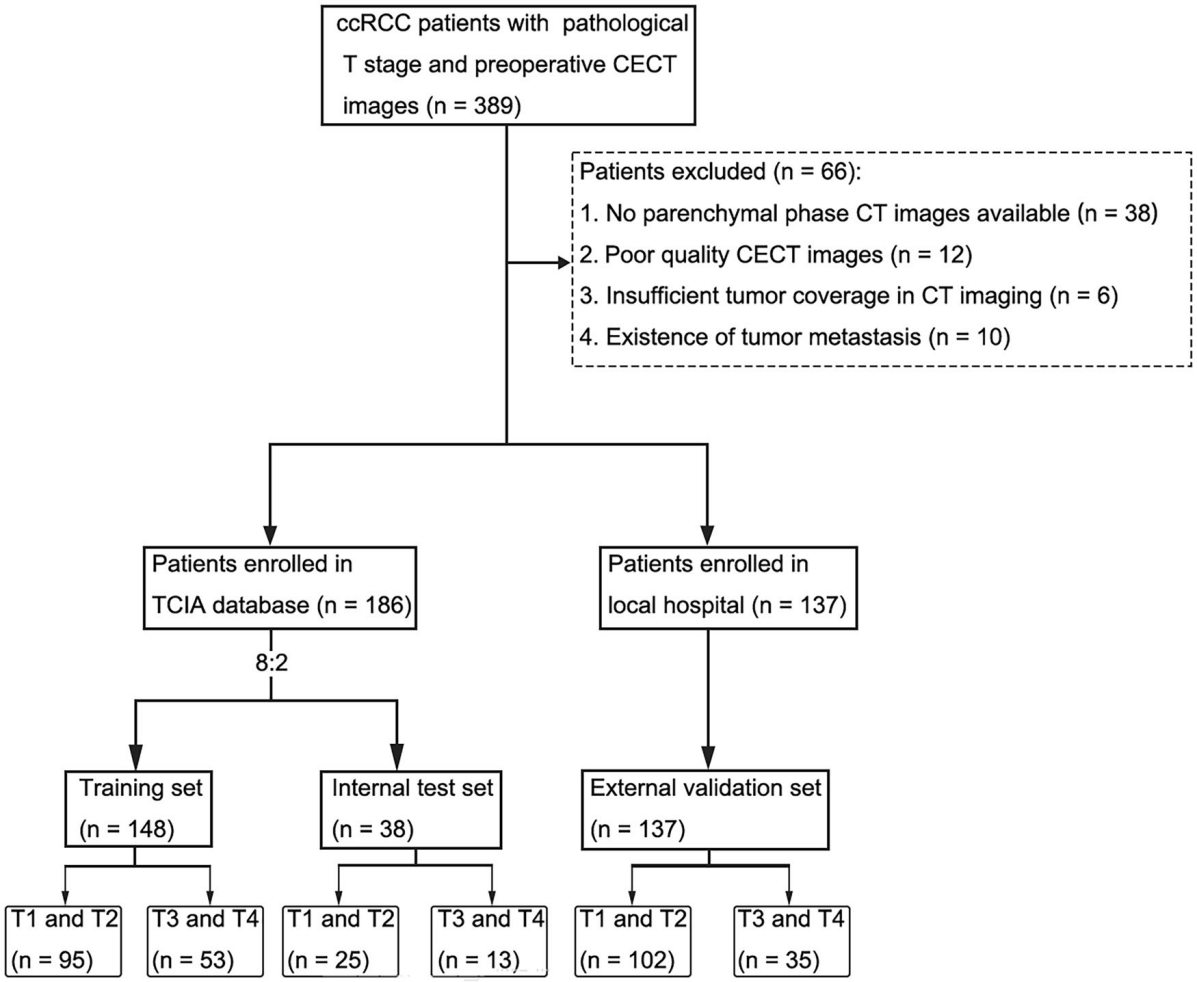
Flowchart of patient recruitment

This was a retrospective study for which approval from the Ethics Committee of the Fourth Hospital of West China, Sichuan University was obtained (No. HXSY-EC-2025096). Informed consents to patients were waived. A total of 323 eligible patients were enrolled from the Cancer Imaging Archive (TCIA) database and the hospital. Specifically, 186 patients from the TCIA database were assigned to the training set (*n* = 148) and the internal test set (*n* = 38). Meanwhile, 137 patients from the hospital, recruited between January 2019 and March 2025, were allocated to the external validation set. The patient recruitment pathway, along with the inclusion and exclusion criteria, is presented in Fig. 1.

Baseline clinicopathological data, including age, sex, laterality, pathological T stage, and follow-up information, were collected. The transcriptomic data corresponding to the TCIA cases were obtained from the Cancer Genome Atlas (TCGA) database. The pathological T stage was assessed in accordance with the International Society of Urological Pathology Consensus recommendations [15]. Patients were divided into two groups based on pathological T stage: the low T stage group (T1 and T2, *n* = 222) and the high T stage group (T3 and T4, *n* = 101).

### Imaging protocol

CT images were acquired using a GE Revolution 256-slice spiral CT scanner at the local hospital. All patients drank 500 mL of water 15 min prior to the examination. For the enhanced scan, a non-ionic contrast agent was administered intravenously via a high-pressure syringe at a dose of 1.5 mL/kg and a flow rate of 2.5 mL/s. Parenchymal phase images from the diaphragm to the kidneys were obtained 60–70 s after contrast injection. The imaging parameters were as follows: matrix size, 512 × 512; rotation time, 0.5 s; tube voltage, 120 kV; tube current, 200–600 mA; detector coverage, 80 mm; pitch, 0.992:1; reconstructed slice thickness and interval, 1.25 mm.

### Radiomics procedure

**Fig. 2:**
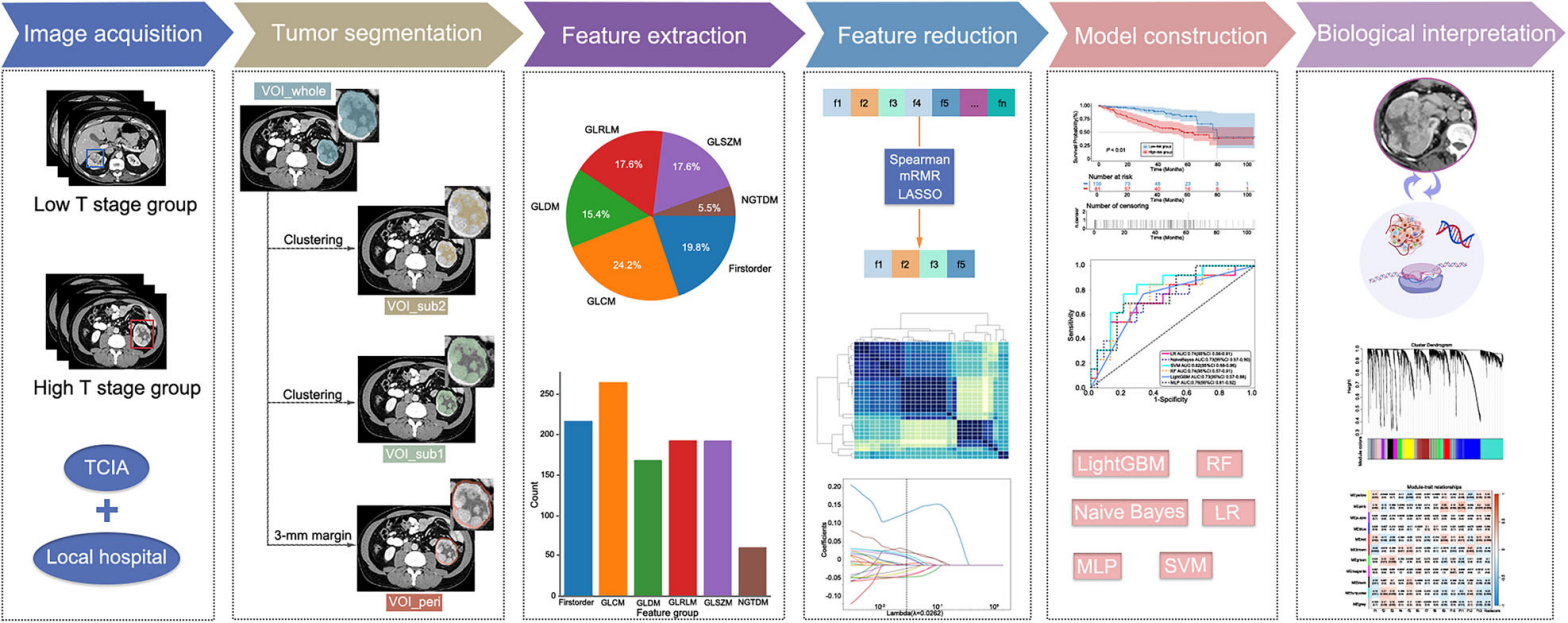
Radiomics analysis workflow

The radiomics analysis involved multiple sequential stages: image acquisition, tumor segmentation, feature extraction, feature reduction, model construction and biological interpretation (Fig. 2).

### VOI segmentation and feature extraction

Volumes of interest (VOIs) of tumors were segmented using 3D Slicer (v5.6.2; Harvard Medical School). Initially, Radiologist A (with 7 years of CT experience) manually delineated the whole tumor region slice-by-slice along the tumor boundary (designated as VOI_whole), which was subsequently validated by Radiologist B (with 20 years of CT experience). Concurrently, the peritumoral region was automatically generated by the software using a 3-mm margin (designated as VOI_peri). To assess inter-observer agreement, both radiologists (RA and RB) independently re-delineated the VOIs on a randomly selected subset of 30 ccRCC patients 1 month later. Intra-class correlation coefficients (ICCs) greater than 0.75 were retained (Supplementary Material S1). The optimal clustering of RFs-homogeneous subregions was achieved using a Gaussian mixture model (GMM), with the cluster quantity (K = 2–4) evaluated using the Bayesian information criterion (BIC) [16, 17].

Standardized image preprocessing was implemented to mitigate heterogeneity arising from multi-scanner CT acquisitions with varying parameters. All datasets underwent isotropic resampling to 1 × 1 × 1 mm³ voxel resolution, coupled with intensity discretization using 25 gray-level bins. PyRadiomics software was used to extract RFs from each region (Fig. S1).

### Feature selection

All RFs underwent Z-score normalization. The feature selection pipeline of each region comprised three sequential stages: First, Spearman correlation analysis identified redundant feature pairs (*p* > 0.9), with one feature retained per pair to mitigate multicollinearity. Second, the maximum relevance minimum redundancy (mRMR) algorithm preserved the top 25 features based on mutual information criteria. Third, the least absolute shrinkage and selection operator (LASSO) regression with penalty parameter tuning via 10-fold cross-validation selected features with nonzero coefficients. Subsequently, the radiomic score (Radscore) was computed as the weighted summation of selected features scaled by their regression coefficients. The optimal risk stratification threshold was determined via the Youden index, maximizing the sum of sensitivity and specificity. Using the optimal cutoff, patients were categorized into high- and low-risk groups.

### Model development and validation

Several machine learning (ML) models were developed using distinct tumor regions: the whole tumor region (VOI_whole), the peritumoral region (VOI_peri), and intratumoral subregions (VOI_subx). Additionally, an optimal intratumoral subregion was combined with the peritumoral region to form the VOI_mix region. Six ML algorithms, including Logistic Regression (LR), Support Vector Machine (SVM), Random Forest (RF), Light Gradient Boosting Machine (LightGBM), Naive Bayes, and Multilayer Perceptron (MLP), were employed for model construction. Model performance was evaluated by the receiver operating characteristic (ROC) curve and the corresponding area under the curve (AUC). The accuracy, precision, recall and F1 score of the models were also calculated.

### Biological mechanisms exploration of radiomics

In our research, the weighted gene co-expression network analysis (WGCNA) was performed to identify modules of highly correlated genes and evaluate module associations with key RFs. Initially, pairwise Pearson correlation coefficients between genes were computed and transformed into a scale-free adjacency matrix using soft-thresholding. Subsequently, a topological overlap matrix (TOM) was applied to refine network connectivity by eliminating spurious edges. Co-expressed genes were then clustered into functional modules via average-linkage hierarchical clustering with a minimum size threshold of 100 genes. Ultimately, the most statistically significant module was identified by correlating module eigengenes with RFs.

Based on this prioritized module, functional annotation was performed: the gene ontology (GO) enrichment analysis for biological processes (BP), cellular components (CC), and molecular functions (MF) was conducted to identify significantly enriched terms. Concurrently, the Kyoto encyclopedia of genes and genomes (KEGG) database was interrogated to pinpoint key signaling pathways, with the top 3 pathways ranked by ascending *p*-value presented.

### Statistical analysis

Statistical analysis was conducted using R software (v3.6.0). Continuous variables, assessed for normality, were expressed as mean ± standard deviation (SD) or median with interquartile range (IQR), as appropriate. Categorical variables were expressed as counts with percentages. Group comparisons employed chi-square or Fisher’s exact tests for categorical data, and independent samples *t*-tests or Mann–Whitney U tests for continuous variables. The association between Radscore and overall survival (OS) was assessed via Kaplan–Meier (KM) analysis, with statistical significance evaluated by the log-rank test. A significance level of *p* < 0.05 was applied throughout the analysis.

## Results

### Baseline characteristics

The clinical characteristics of patients in the training set, internal test set, and external validation set are shown in Table 1. In the training and internal test sets, there were no statistically significant differences in sex, age, and laterality between the high T stage group and the low T stage group (*p* > 0.05). A significant difference in sex distribution was observed in the external validation set (*p* < 0.05); however, this should be interpreted with caution as it was not adjusted for multiple testing.

**Table 1:** Clinical information of this study

Feature	Training set (*n* = 148)		*p*-value	Internal test set (*n* = 38)		*p*-value	External validation set (*n* = 137)		*p*-value
	High T stage group (*n* = 53)	Low T stage group (*n* = 95)		High T stage group (*n* = 13)	Low T stage group (*n* = 25)		High T stage group (*n* = 35)	Low T stage group (*n* = 102)	
Age (years)	61.60 ± 10.87	59.11 ± 12.04	0.21	63.46 ± 11.33	54.84 ± 13.26	0.05	56.91 ± 10.49	55.73 ± 11.31	0.59
Sex, *n* (%)			0.15			1			0.01*
Male	31 (58.49%)	68 (71.58%)		8 (61.54%)	16 (64.00%)		17 (48.57%)	75 (73.53%)	
Female	22 (41.51%)	27 (28.42%)		5 (38.46%)	9 (36.00%)		18 (51.43%)	27 (26.47%)	
Laterality, *n* (%)			0.83			0.17			0.53
Right	28 (52.83%)	47 (49.47%)		10 (76.92%)	12 (48.00%)		21 (60.00%)	53 (51.96%)	
Left	25 (47.17%)	48 (50.53%)		3 (23.08%)	13 (52.00%)		14 (40.00%)	49 (48.04%)	

* Statistically significant difference

### Subregion cluster and feature selection

**Fig. 3:**
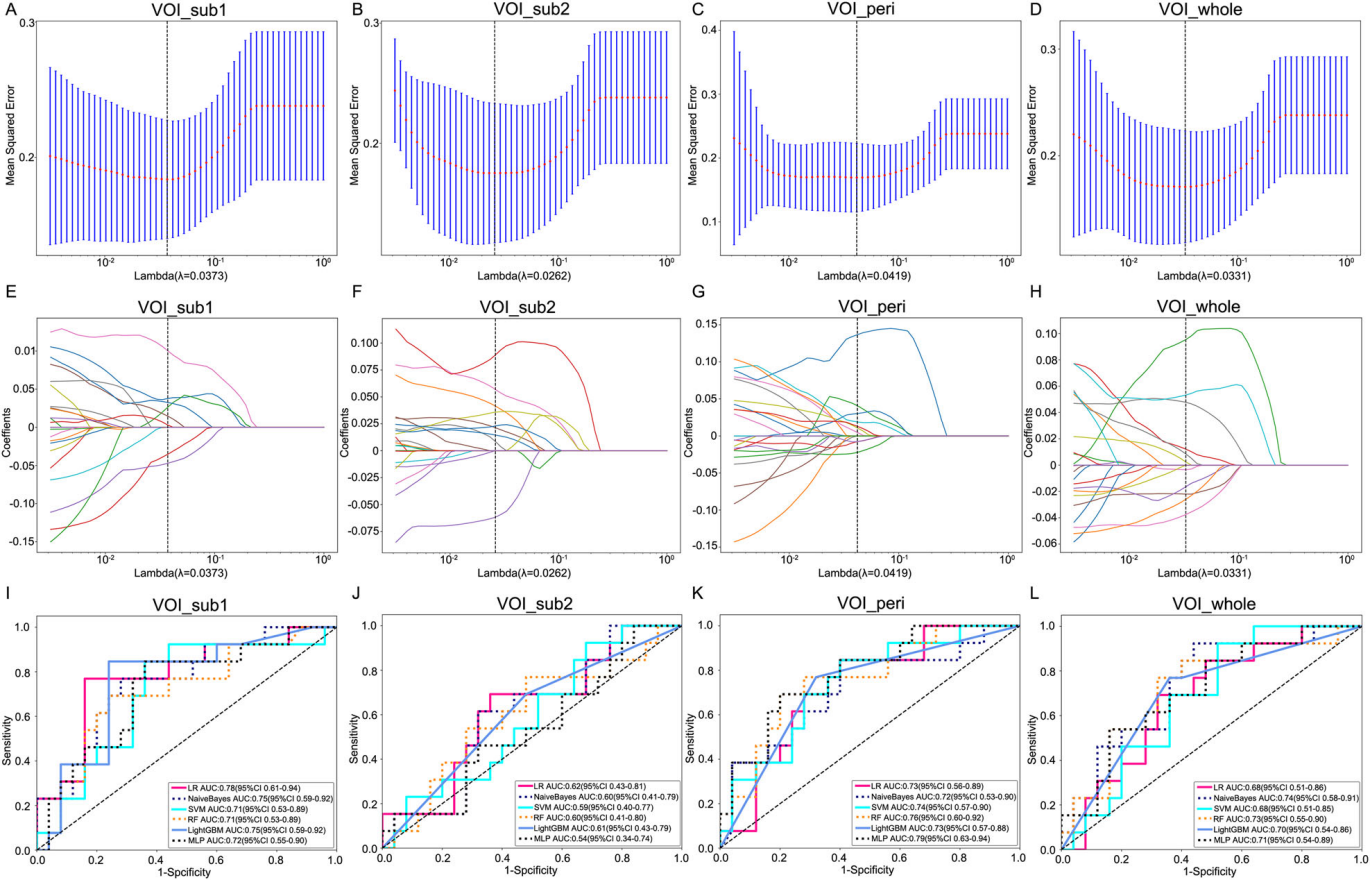
LASSO feature selection and model validation. **A**–**D** Selection of tuning parameter (λ) in the LASSO model. The vertical line presented the optimal λ value. **E**–**H** Plot of the LASSO coefficient profiles of the selected features. The vertical line presented the optimal λ value. **I**–**L** ROC curves of models based on VOI_sub1, VOI_sub2, VOI_peri and VOI_whole in the test set

The GMM analysis identified an optimal cluster count of 2 (Fig. S2), prompting division of the VOI_whole into two distinct subregions (VOI_sub1 and VOI_sub2) (Fig. S3). From each of the four regions (VOI_sub1, VOI_sub2, VOI_peri, and VOI_whole), 1093 RFs were extracted, yielding a total of 4372 features. After LASSO analysis, 8, 9, 10 and 11 RFs with nonzero correlation coefficients were identified for developing the ML models, respectively (Fig. 3A–H).

### Model performance and Radscore construction

**Fig. 4:**
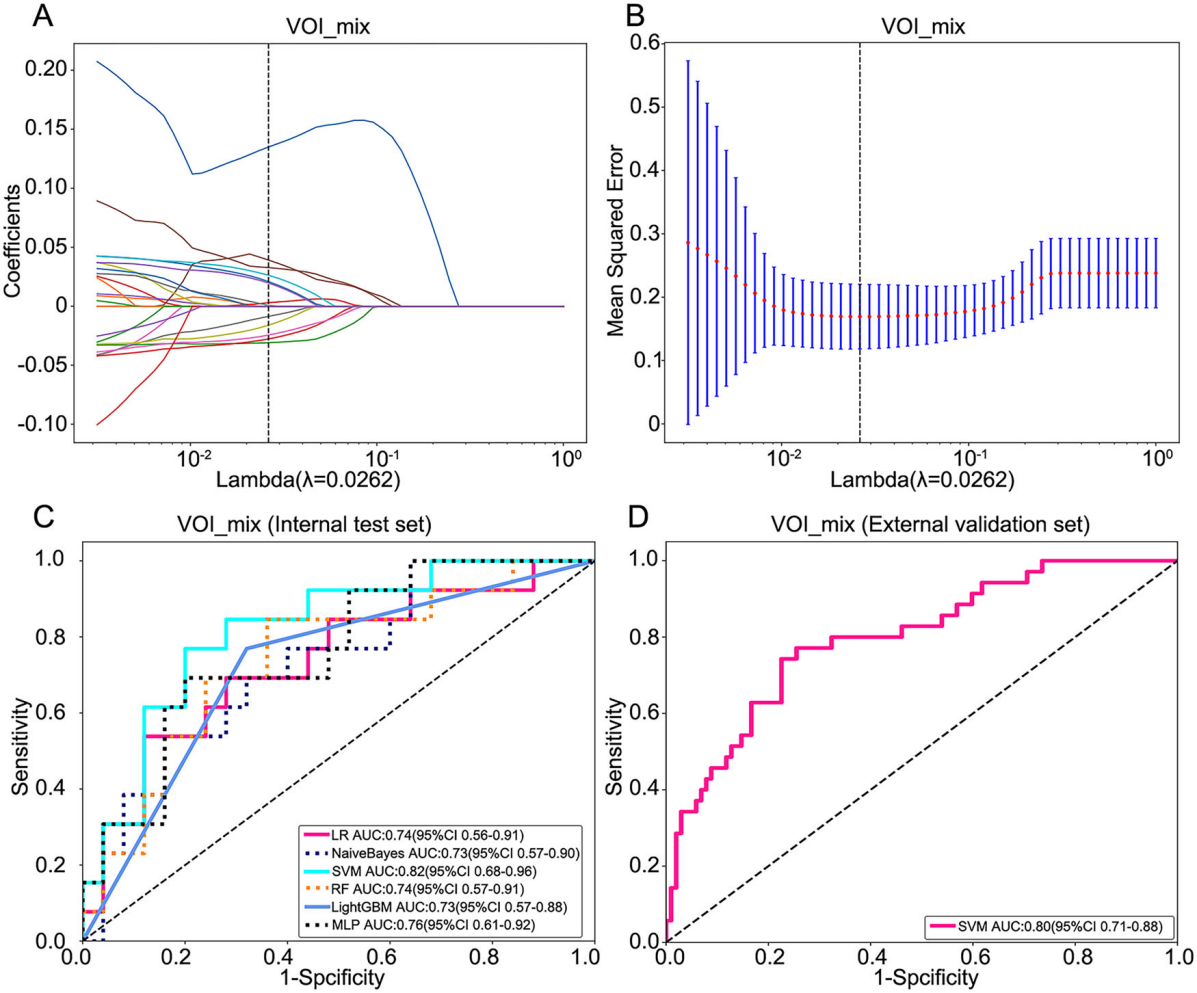
Combined model development and validation. **A** LASSO coefficient path for the 13 selected features from the combined VOI_mix. **B** Cross-validated MSE versus log(λ) used to determine the optimal penalty. **C**, **D** ROC curves of the SVM-based combined model achieving AUCs of 0.82 (95% CI 0.68–0.96) in the internal test set and 0.80 (95% CI 0.71–0.88) in the external validation set

The diagnostic performance of each model for predicting pathological T stage of ccRCC is shown in Table 2 and Fig. 3I–L. Among all models, the MLP model based on VOI_peri and the LR model based on VOI_sub1 demonstrated superior predictive performance, achieving AUC values of 0.79 (95% CI: 0.63–0.94) and 0.78 (95% CI: 0.61–0.94), respectively, in the internal test set. Consequently, we integrated RFs from these two regions (VOI_mix) to develop a combined model based on 13 predictive features after lasso analysis (Fig. 4A, B). The SVM-based combined model achieved the highest AUC values, of 0.82 (95% CI: 0.68–0.96) in the internal test set and 0.80 (95% CI: 0.71–0.88) in the external validation set (Table 3, Fig. 4C, D).

**Table 2:** Prediction performance of models in the internal test set

Model	Accuracy	Precision	Recall	F1-score	AUC (95% CI)
VOI_whole					
LR	0.68	0.53	0.69	0.60	0.68 (0.51–0.86)
NaiveBayes	0.68	0.52	0.92	0.67	0.74 (0.57–0.91)
SVM	0.63	0.48	0.92	0.63	0.68 (0.51–0.85)
RF	0.71	0.56	0.77	0.65	0.73 (0.55–0.90)
LightGBM	0.68	0.53	0.77	0.65	0.70 (0.54–0.86)
MLP	0.74	0.64	0.54	0.58	0.71 (0.54–0.87)
VOI_sub1					
LR	0.82	0.71	0.77	0.74	0.78 (0.61–0.94)
NaiveBayes	0.74	0.59	0.77	0.67	0.75 (0.59–0.91)
SVM	0.71	0.55	0.85	0.67	0.71 (0.53–0.89)
RF	0.74	0.60	0.69	0.64	0.71 (0.53–0.89)
LightGBM	0.79	0.65	0.85	0.73	0.75 (0.59–0.92)
MLP	0.71	0.55	0.85	0.67	0.72 (0.55–0.90)
VOI_sub2					
LR	0.66	0.50	0.69	0.58	0.62 (0.43–0.81)
NaiveBayes	0.66	0.50	0.62	0.55	0.60 (0.41–0.79)
SVM	0.53	0.41	0.92	0.57	0.59 (0.40–0.77)
RF	0.61	0.46	0.77	0.57	0.60 (0.41–0.80)
LightGBM	0.58	0.43	0.69	0.53	0.61 (0.43–0.79)
MLP	0.45	0.38	1.00	0.55	0.54 (0.34–0.74)
VOI_peri					
LR	0.68	0.52	0.85	0.65	0.73 (0.56–0.89)
NaiveBayes	0.68	0.52	0.85	0.65	0.72 (0.53–0.90)
SVM	0.68	0.52	0.85	0.65	0.74 (0.57–0.90)
RF	0.76	0.64	0.69	0.67	0.76 (0.60–0.92)
LightGBM	0.71	0.56	0.77	0.65	0.73 (0.57–0.88)
MLP	0.76	0.64	0.69	0.67	0.79 (0.63–0.94)

*AUC* area under the curve, *LR* logistic regression, *SVM* support vector machine, *RF* random forest, *LightGBM* Light Gradient Boosting Machine, *MLP* multilayer perceptron

**Table 3:** Prediction performance of models in the internal test and external validation sets

Model	Internal test set					External validation set				
	Accuracy	Precision	Recall	F1-score	AUC (95% CI)	Accuracy	Precision	Recall	F1-score	AUC
VOI_mix										
LR	0.76	0.70	0.54	0.61	0.74 (0.56–0.91)	0.73	0.48	0.77	0.59	0.81 (0.73–0.88)
NaiveBayes	0.74	0.64	0.54	0.58	0.73 (0.57–0.90)	0.76	0.53	0.51	0.52	0.72 (0.62–0.82)
SVM	0.79	0.67	0.77	0.71	0.82 (0.68–0.96)	0.77	0.53	0.74	0.62	0.80 (0.71–0.88)
RF	0.71	0.55	0.85	0.67	0.74 (0.57–0.91)	0.76	0.52	0.80	0.63	0.83 (0.76–0.90)
LightGBM	0.71	0.56	0.77	0.65	0.73 (0.57–0.88)	0.70	0.45	0.80	0.58	0.73 (0.65–0.81)
MLP	0.76	0.64	0.69	0.67	0.76 (0.61–0.92)	0.79	0.58	0.63	0.60	0.80 (0.72–0.88)

*AUC* area under the curve, *LR* logistic regression, *SVM* support vector machine, *RF* random forest, *LightGBM* Light Gradient Boosting Machine, *MLP* multilayer perceptron

**Fig. 5:**
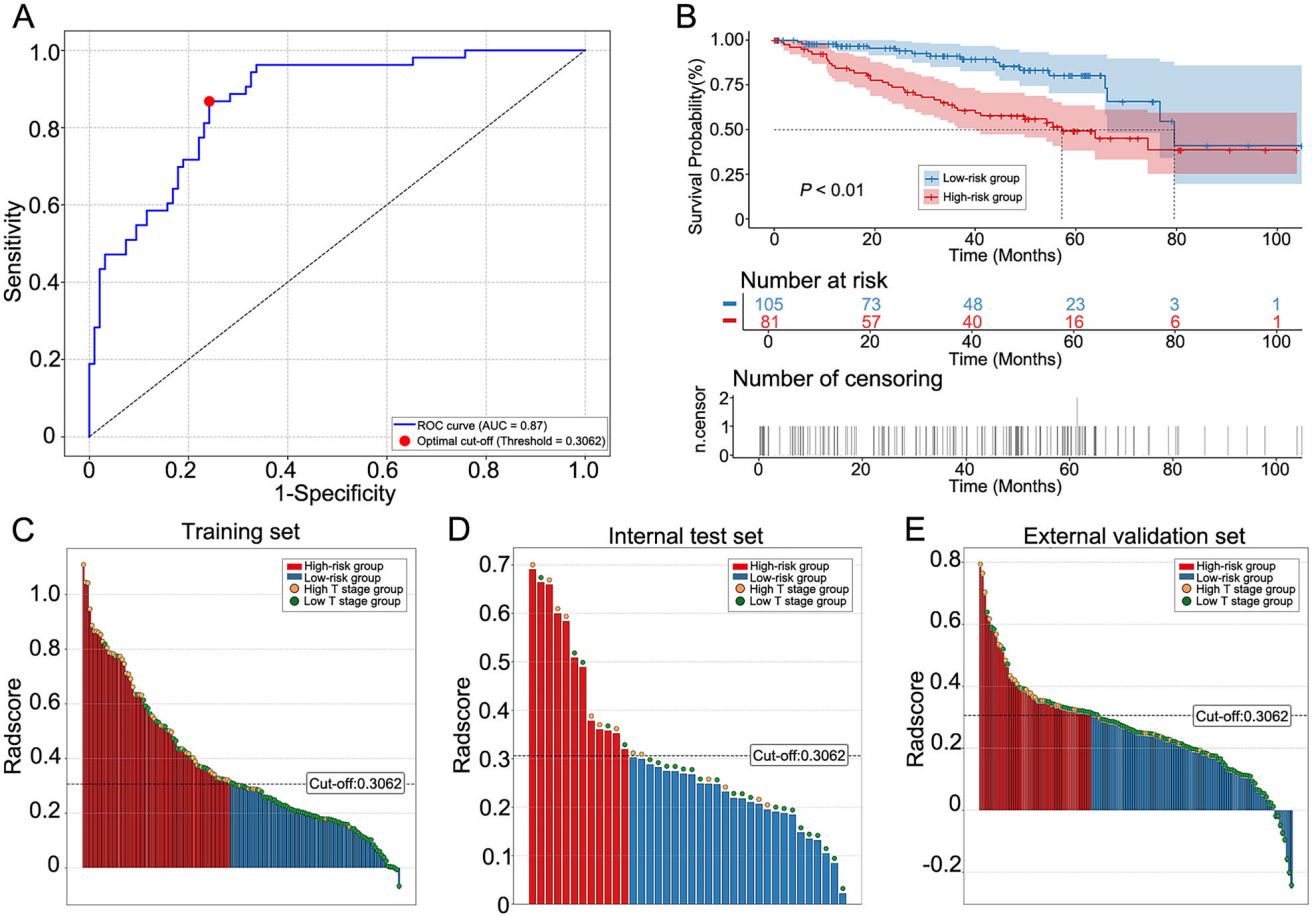
Radscore-based risk stratification and survival analysis. **A** Optimal Radscore cutoff (0.3062) determination by the Youden index. **B** Kaplan–Meier analysis of overall survival in patients with ccRCC stratified by Radscore (high-risk vs. low-risk groups). **C**–**E** Optimal cutoff-based Radscore distribution and risk stratification in the training, internal test and external validation cohorts

Based on these 13 RFs and their corresponding coefficients derived from the LASSO model, we computed a Radscore as a linear combination of the selected features weighted by their respective LASSO coefficients (Supplementary Material S2). The optimal cutoff value for the Radscore was determined to be 0.3062 (Fig. 5A). All patients were categorized into a high-risk group (Radscore ≥ 0.3062) and a low-risk group (Radscore < 0.3062) (Fig. 5C–E). KM curves demonstrated that OS was significantly lower in the high-risk group than in the low-risk group (*p* < 0.01) (Fig. 5B).

### Association between RFs and biological functions

**Fig. 6:**
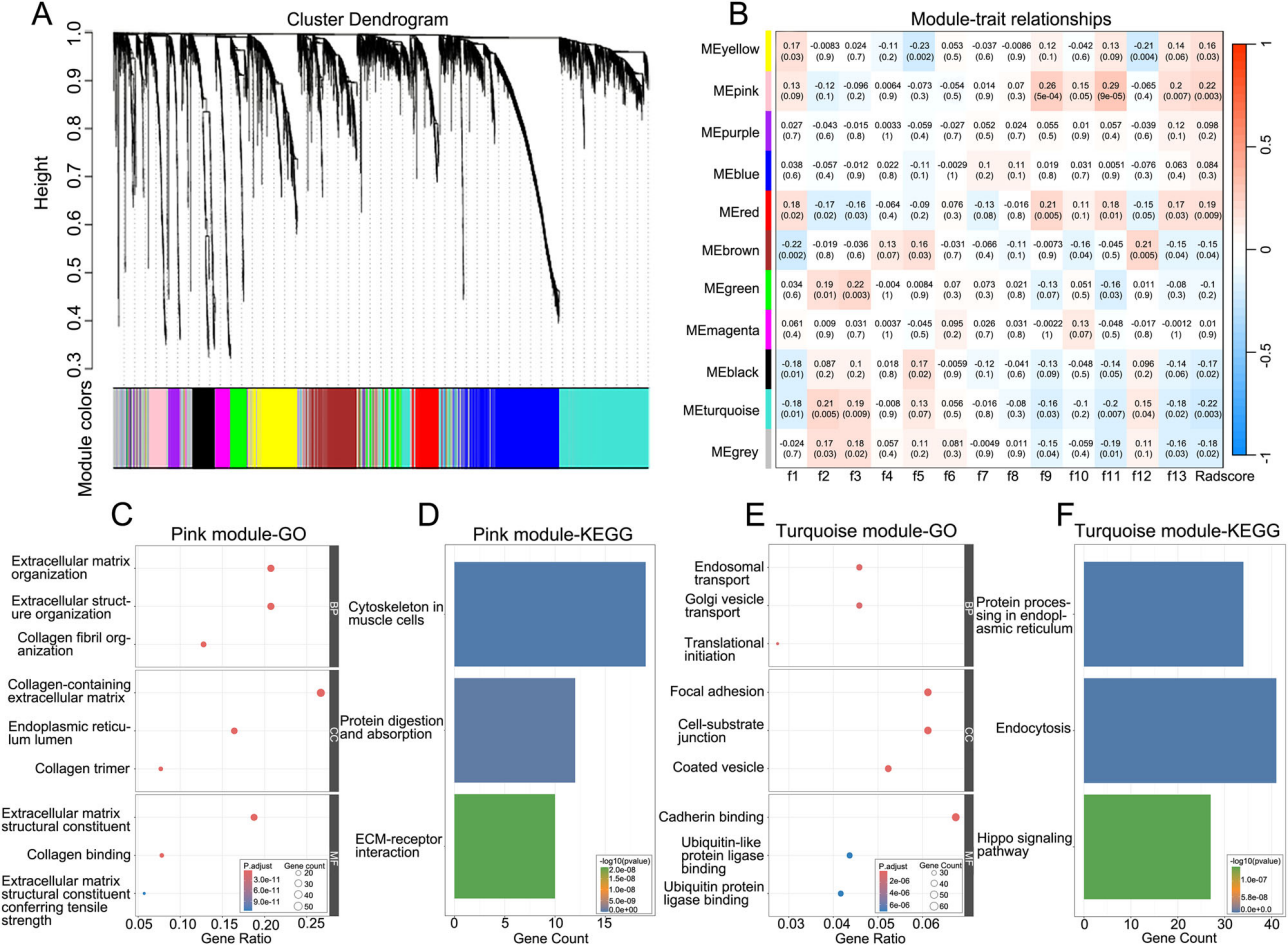
The exploration of molecular biological mechanisms of the RFs. **A** Dendrogram of gene modules identified by WGCNA (11 modules shown in distinct colors). **B** Heatmap of module–radiomics correlations. The pink module (r = 0.22, *p* = 0.003) and turquoise module (r = −0.22, *p* = 0.003) displayed the most significant correlation. **C**, **E** GO enrichment analysis of genes in the pink and turquoise module, including biological processes (BP), cellular components (CC) and molecular functions (MF) categories. **D**, **F** KEGG pathway analysis of genes in the pink and turquoise module

In this study, the soft-threshold power was set at 5. The WGCNA analysis identified 11 gene modules (Fig. 6A). Notably, the pink module (r = 0.22, *p* = 0.003) and the turquoise module (r = −0.22, *p* = 0.003) exhibited strong positive and negative correlations with the Radscore, respectively (Fig. 6B). Given their significant associations with the Radscore, these two modules were selected for GO and KEGG analysis.

GO analysis revealed that the pink module was significantly enriched in biological functions including collagen fibril organization (BP), collagen-containing extracellular matrix (CC), and extracellular matrix structural constituent (MF) (Fig. 6C). In contrast, the turquoise module primarily exhibited enrichment in endosomal transport (BP), coated vesicle (CC), and cadherin binding (MF) (Fig. 6E). KEGG pathway analysis further demonstrated significant enrichment of the pink module in cytoskeleton in muscle cells, protein digestion and absorption and ECM-receptor interaction (Fig. 6D), while the turquoise module was enriched in protein processing in endoplasmic reticulum, endocytosis and Hippo signaling pathway (Fig. 6F).

## Discussion

The pathological T stage of ccRCC primarily relied on postoperative pathological diagnosis, while accurate preoperative prediction of the pathological T stage was crucial for individualized treatment planning. However, there is currently a lack of non-invasive methods in clinical practice that can accurately predict the preoperative pathological T stage of ccRCC patients. In light of this, we constructed an ML model to predict the pathological T stage non-invasively via a subregional and peritumoral radiomic method. This model demonstrated good predictive performance in both the internal test set and the external validation set, with AUC values reaching 0.82 and 0.80, respectively. Moreover, by integrating multi-omics data, the study further explored the underlying biological mechanisms of radiomics, providing important evidence for the biological interpretation of RFs and facilitating the translation of radiomics into clinical practice.

Some studies have investigated the value of radiomics in predicting ccRCC TNM stage. For example, Demirjian et al [10] developed radiomic models that achieved the AUCs of 0.80 and 0.77 in the full model and robust model to distinguish between low- and high-stage ccRCC. Ökmen et al [18] constructed a radiomic classification learner that achieved an overall accuracy of 85.4% in predicting TNM stages. However, conventional radiomic approaches have largely treated tumors as homogeneous entities, potentially overlooking critical intratumoral heterogeneity. Our study addressed this limitation by employing k-means clustering to segment tumors into two distinct intratumoral subregions, enabling a more comprehensive characterization of spatial heterogeneity [19–22]. Notably, the model based on VOI_sub1 demonstrated superior predictive performance compared to whole-tumor analysis (AUC: 0.78 vs. 0.74), suggesting that this subregion may harbor biologically aggressive features not captured by global tumor assessment.

The tumor microenvironment played a vital role in regulating malignant biological behaviors, including tumor growth, invasion and metastasis, immune evasion, and angiogenesis [23–27]. While previous studies have demonstrated the value of peritumoral RFs in predicting tumor invasiveness [28], metastasis [29], response to therapy [30], and prognosis assessment [31], their application in predicting the pathological T stage of ccRCC has not been reported. In this study, we innovatively combined RFs from a 3-mm peritumoral region with intratumoral subregion analysis. The resulting combined prediction model achieved the highest predictive accuracy (AUC: 0.82).

Based on 13 key features, the Radscore effectively stratified the prognosis of ccRCC patients. High-risk patients had significantly worse OS than low-risk patients. This suggested that the Radscore could help identify aggressive tumors early, enabling more intensive treatment strategies to improve outcomes. The limited interpretability of radiomics hindered its clinical translation. Through radiogenomics analysis, this study elucidated the underlying biological significance of radiomics. The elevation of Radscore was significantly positively correlated with extracellular matrix remodeling, such as collagen fiber organization and extracellular matrix-receptor interactions, which provided a structural basis for tumor invasion [32–34]. Conversely, it was significantly negatively correlated with intracellular processes, including vesicle transport, protein processing in the endoplasmic reticulum, and inhibition of the Hippo signaling pathway, which collectively drove tumor cell proliferation and migration [35–37]. These findings suggested that RFs could capture the key molecular mechanisms underlying tumor invasiveness and hold potential as non-invasive biomarkers for assessing the biological invasiveness of clear ccRCC.

This study has several limitations. First, the data distribution between the low T-stage and high T-stage groups was imbalanced, which may have affected the accuracy of the model in predicting certain T stages. Although we employed class weight balancing during model training to mitigate this, future studies with larger, more balanced cohorts are warranted to validate our findings. Second, differences in CT scanning protocols across various machines may have impacted the stability of RFs extraction and analysis. Although we applied standardized image preprocessing to mitigate this heterogeneity, we did not employ advanced harmonization techniques like ComBat. The potential influence of scanner variability on our model’s performance should be considered, and future multi-center studies with dedicated harmonization protocols are encouraged. Third, this study only included parenchymal phase images, and future research should incorporate non-contrast and other enhanced phase images to provide more comprehensive information. Lastly, the association analysis between RFs and biological pathways was still in its preliminary stage. The radiogenomic correlations identified here require functional validation through in vitro or in vivo experiments to establish causality. More in-depth research is required to further elucidate the biological mechanisms and clinical relevance of these features.

## Conclusion

This study developed a preoperative pathological T-stage prediction model for ccRCC using subregional and peritumoral radiomics analysis, achieving good performance in internal and external validation. The Radscore derived from RFs effectively stratified patients for prognosis. By integrating multi-omics data, the study further explored the biological mechanisms of RFs and revealed their potential links to tumor biological processes. These findings provided a new method for the non-invasive diagnosis and prognostic assessment of ccRCC and laid the foundation for future research and clinical applications.

## Supplementary information

ELECTRONIC SUPPLEMENTARY MATERIAL

Supplementary information

## Data Availability

Summary data from the survey are presented within the manuscript and accompanying figures. Applicable hyperlinks to publicly archived datasets: (1) TCIA: https://www.cancerimagingarchive.net/; (2) TCGA: https://portal.gdc.cancer.gov/.

## Abbreviations

BP: Biological process
CC: Cellular component
ccRCC: Clear cell renal cell carcinoma
ECM: Extracellular matrix
GMM: Gaussian mixture model
GO: Gene ontology
ICC: Intra-class correlation coefficient
KEGG: Kyoto Encyclopedia of Genes and Genomes
KM: Kaplan–Meier
LASSO: Least Absolute Shrinkage and Selection Operator
LightGBM: Light Gradient Boosting Machine
LR: Logistic regression
MF: Molecular function
ML: Machine learning
MLP: Multilayer perceptron
OS: Overall survival
Radscore: Radiomic score
RCC: Renal cell carcinoma
RF: Random Forest
RFs: Radiomic features
SVM: Support vector machine
TCIA: The Cancer Imaging Archive
TNM: Tumor node metastasis
VOI: Volume of interest
VOI_mix: Combined Subregion and Peritumoral Region
VOI_peri: Peritumoral region
VOI_subx: Intratumoral subregions
VOI_whole: Whole tumor region
WGCNA: Weighted gene co-expression network analysis
