# Supplementary material for: CT-based subregional and peritumoral radiomics for predicting pathological T stage of clear cell renal cell carcinoma: an exploratory study of biological mechanisms
Source: Insights Imaging. 2026 Feb 16;17:50. doi: 10.1186/s13244-026-02226-3 (PMC12909736; doi:10.1186/s13244-026-02226-3)
Supplement: Supplementary file 1 — ELECTRONIC SUPPLEMENTARY MATERIAL [file 13244_2026_2226_MOESM1_ESM.pdf]

CT-based subregional and peritumoral radiomics for predicting pathological T stage of clear cell renal cell carcinoma: an exploratory study of biological mechanisms

ELECTRONIC SUPPLEMENTARY MATERIAL

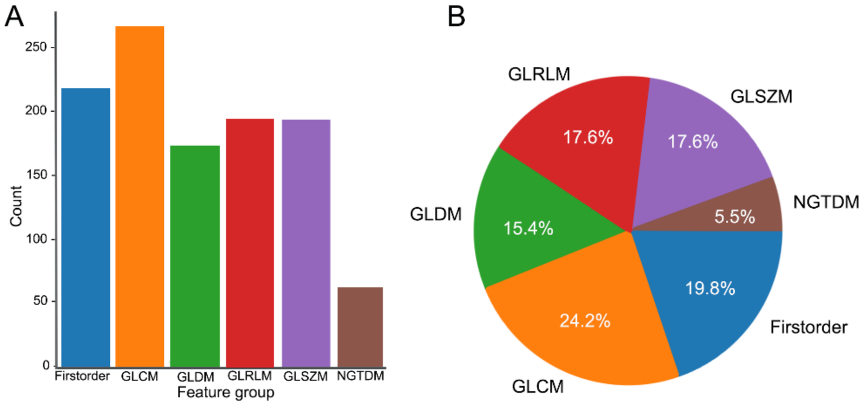

Fig. S1 The radiomic features extracted from each region. **A-B** The features were grouped into the following categories: Firstorder, n = 216; grey-level cooccurrence matrix (GLCM), n = 264; grey-level run length matrix (GLRLM), n = 192; grey-level size zone matrix (GLSZM), n = 192; grey-level dependence matrix (GLDM), n = 168; and neighbouring grey tone difference matrix (NGTDM), n = 60.

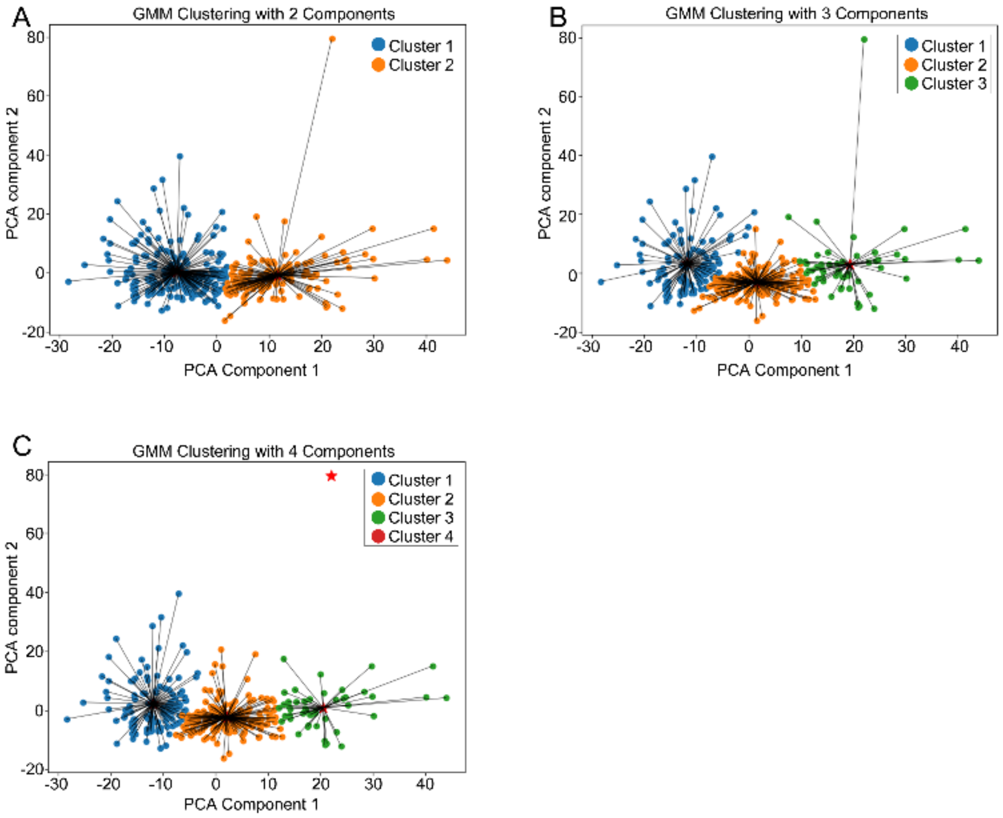

Fig. S2 Gaussian mixture model (GMM) clustering of radiomics feature using principal component analysis (PCA)-reduced data. **A-C** the clustering results for 2, 3, and 4 components, respectively. Points represent samples, colored by cluster. Red stars indicate cluster centroids.

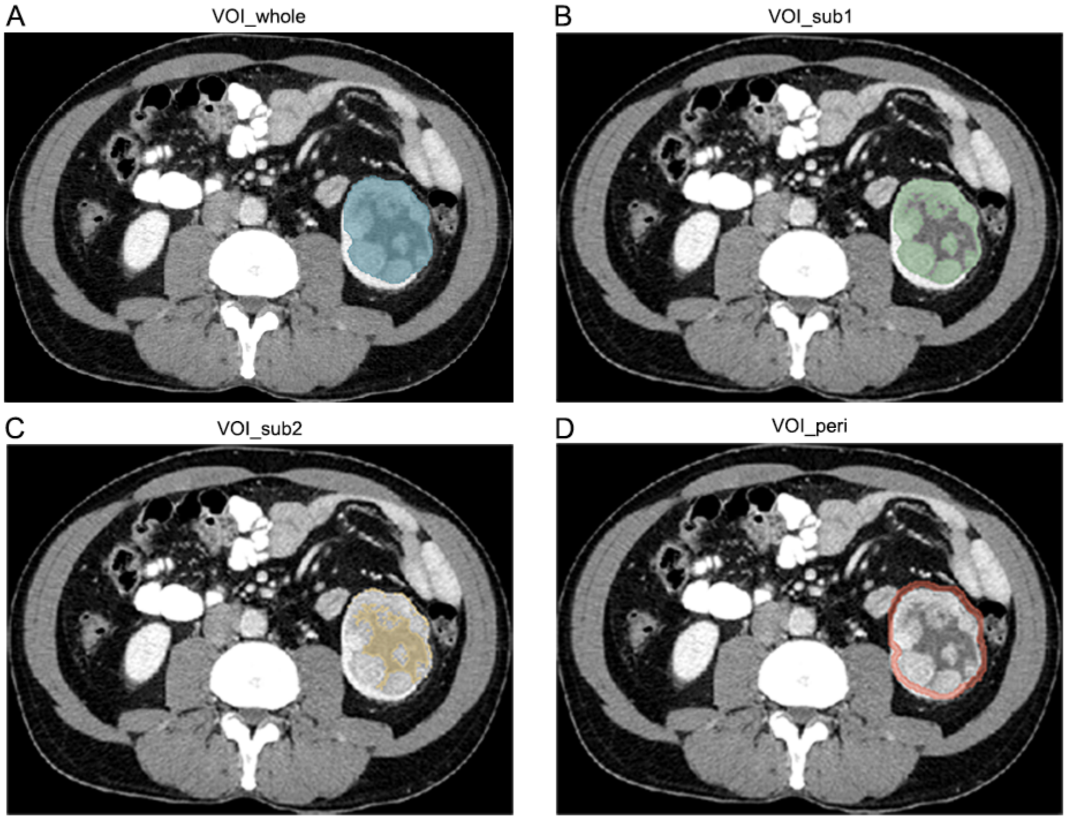

Fig. S3 Examples of volumetric segmentation for intratumoral subregions and the peritumoral region. **A** Whole tumor volume (VOI_whole). **B** Intratumoral subregion 1 (VOI_sub1), predominantly representing the viable tumor core. **C** Intratumoral subregion 2 (VOI_sub2), predominantly representing the necrotic/cystic component. **D** Peritumoral region (VOI_peri) with a 3-mm margin.VOI, volume of interest.

Supplementary Material 2:

Radscore = b + a1X1 + a2X2 + a3X3+......+aiXi, where Xi represents the selected RF, ai is the regression coefficient of the corresponding RF, and b is the intercept. The calculation formula of the radscore was as follows:

Radscore = 0.35810810810810934 + 0.002974 * f1-0.027719 * f2-0.008586 * f3-0.016029 * f4-0.024379 * f5+0.019657 * f6+0.020852 * f7+0.026286 * f8+0.039124 * f9+0.000711 * f10+0.032754 * f11-0.030888 * f12+ 0.134954 * f13

f1:log_sigma_2_0_mm_3D_glcm_Idn_h1

f2:original_ngtdm_Strength_h1

f3:wavelet_LLL_ngtdm_Strength_h1

f4:original_glszm_LargeAreaLowGrayLevelEmphasis_h2

f5:original_glszm_SmallAreaLowGrayLevelEmphasis_h2

f6:wavelet_HHL_glcm_ClusterProminence_h2

F7:wavelet_HLH_firstorder_RootMeanSquared_h2

f8:wavelet_HLL_glcm_ClusterProminence_h2
f9:wavelet_LHH_gldm_GrayLevelNonUniformity_h2

f10:wavelet_LHL_firstorder_Kurtosis_h2

f11:wavelet_LLL_glszm_GrayLevelNonUniformity_h2
f12:wavelet_LLL_glszm_LargeAreaLowGrayLevelEmphasis_h2

f13wavelet_LLL_glszm_SizeZoneNonUniformity_h2
